# Fluid responsiveness is about stroke volume, and not pulse pressure Yogi: the power of Doppler fluid management and cardiovascular monitoring

**DOI:** 10.1007/s10877-014-9598-y

**Published:** 2014-07-22

**Authors:** Rob Phillips, Joe Brierley

**Affiliations:** 1 The School of Medicine, The University of Queensland, Brisbane, Australia; 2 Director of Clinical Science, Uscom Limited, Level 7/10 Loftus St, Sydney, 2000 NSW Australia; 3 PICU, Great Ormond St Hospital for Children and University College, London, United Kingdom

**Keywords:** Advanced haemodynamics, Doppler, Monitoring, Fluid responsiveness, Blood pressure, PPV

## Abstract

Fluid infusion is one of the most common critical care interventions, yet approximately 50 % of all fluid interventions are unnecessary and potentially harmful. An improved approach to identification of fluid responsiveness is of clinical importance. Currently fluid responsiveness is most frequently identified by blood pressure (BP) measurements or a surrogate. However fluid responsiveness is simply the increase in stroke volume (SV) associated with volume expansion, and may not be reflected in BP or BP surrogates. Guyton demonstrated that BP = CO x SVR, and it is know that baroreceptor mediated autonomic nervous system regulation of SV and SVR to preserve BP may mask significant and critical changes in haemodynamics. Dr Pinsky in his recent J Clin Monit Comput Editorial evaluated the relative merits of pulse pressure variability (PPV) methods, a variant on BP measurement, for assessment of fluid responsiveness and promoted the use of physiologic challenges to augment the applicability of PPV. However this guidance is only half right. This letter reminds clinicians of the physiologic limitations of PPV as a measure of fluid responsiveness, even when combined with physiologic challenges, and recommends the replacement of BP with SV measurements. The combination of accurate Doppler measurement of SV and physiologic challenges, as Dr Pinsky recommends, is a physiologically rational and effective approach to identification of fluid responsiveness with established evidence. The direct monitoring of SV and SV changes has the potential to improve a long standing critical care and anaesthetic conundrum; when to give fluid and when to stop.

## Introduction

While we have the utmost respect for both the great baseball philosopher Yogi Berra and Michael Pinsky, it would seem that in Pinsky’s recent Editorial “It is amazing what you can see if you look” on fluid responsiveness and physiologic challenges, we have been provided with only half the clues [1].

Dr Pinsky’s central point is that physiologic challenges improve the effectiveness and applicability of PPV monitoring for identifying fluid responsiveness, and this is incontestable. However basic physiology ultimately limits the effectiveness of PPV monitoring for identification of fluid responsiveness as Benes and others have observed [2, 3].

This editorial raises two questions related to physiology. Does PPV approximate SVV, and is the change in PPV–dPPV, equivalent to the change in SV–dSV, the definitive measure of fluid responsiveness as demonstrated by Frank and Starling?

While detection of fluid responsiveness by PPV is limited to approximately 2–51 % of ICU patients [2, 3], Pinsky suggests that dPPV before and after a dynamic physiologic challenge can improve this applicability. This may be possible, but we would argue that this is an unnecessarily complicated approach that appears designed to compensate for the fundamental physiologic failings of PPV rather than to simplify and improve the clinical process of identifying fluid responsiveness.

Despite being a common intervention and clinical conundrum, fluid responsiveness is defined quite simply; fluid responsiveness is a change in SV associated with volume expansion. Yet current methods of assessing fluid responsiveness are predominantly based on surrogates measures of BP—MAP, CVP, IVCd, RAP, PCWP and now PPV. This persistent use of BP and its analogues, such as PPV, to guide fluid therapy, may explain the persistent observation that 50 % or more of all fluid infusions are unnecessary, and potentially harmful [4].

No matter how hard we look at BP or its pressure surrogates, with any number of trials of selected patient cohorts, using any number of devices attached to fingers, or inserted in arteries, and during whatever physiologic challenges, ultimately the baroreceptor modulated Autonomic Nervous System (ANS) ensures that we only see the BP, and not the SV response!

Guyton demonstrated that BP = CO × SVR, thus any correlation between BP (PPV) and CO (SVV) is dependent on SVR remaining constant. While PPV may show some correlation with SVV and fluid responsiveness in normal stable patients, the dynamic beat to beat regulation of ventriculo-arterial coupling by the ANS precludes its application in the dynamic circulation of sick patients. BP, the product of CO(SV) and SVR, is effectively preserved in a baroreceptor determined range by the ANS, so even substantial derangements of either CO/SV or SVR/Ea, will be paradoxically regulated by the ANS to preserve BP. Especially in children, such compensation can effectively mask significant underlying abnormalities of CO/SV and or SVR which may require therapy [5]. If BP is used as a guide, then appropriate therapy isn’t initiated until decompensation and hypotensive shock occurs. This eventual fall in BP is a sign that the ANS, and most likely the physician, has failed, and explains the high mortality associated with hypotensive shock!

This dynamic modulation of BP complicates the relationship between PPV and SVV, and prevents PPV from accurately tracking CO(SV) and SVR, and thereby identifying cardiovascular abnormalities discrete from BP. This ensures that PPV is both a poor guide for fluid responsiveness, and an ineffective guide for inotropes and vaso-active therapies.

From a physiologic stand point it is elementary that the accurate measurement of SV is fundamental for the reliable identification of fluid responsiveness, and that BP measures, including PPV, are therefore only useful insofar as they correlate with SV or SVV, or those in whom ANS regulation is relatively inactive.

Whilst Pinsky may challenge the retrospective methodology of Benes’ study, physiology, animal research and other clinical studies support these findings of limited PPV applicability.

In 2013 Bouchacourt et al. studied dynamic arterial pressure surrogates, including PPV, to track SVV changes during progressive induced hypovolemia in a rabbit model of hemorrhage while increasing vasomotor tone with phenylephrine [6]. They found that dynamic arterial pressure indices, including PPV, were ineffective for detecting fluid responsiveness, and did not reflect the extent of fluid loss compared to SVV as the gold standard. This can be explained by the ANS effectively up-regulating the SVR in response to the decreased SV induced by the hypovolemia, to preserve the BP, or BP ≠ CO(SV) as Guyton observed.

Though PPV may have some application in detection of fluid responsiveness in normal patients, the presence of any source of disturbed or dynamic ventriculo-arterial coupling, such as arrhythmia, vaso-active therapies, irregular or complex respiration, and of course diseases with dynamic effects on cardiac and vascular function, such as sepsis, further inhibits the effectiveness and application of PPV [2, 7]. In short the types of patients that require high level haemodynamic monitoring are precisely the ones in whom PPV is least effective. While the addition of autologous challenges may improve the 2-51 % feasibility reported by Benes et al. and Mahjoub et al. [2, 3], PPV will remain a physiologically ineffective method of detecting fluid responsiveness in the majority of sick patients.

The limits of isolated pressure-based circulatory management have been further highlighted by Asfar et al. [8] in a recent randomised control trial demonstrating that targeting either a high or low BP range in septic shock patients produced no significant difference in outcomes. The ProCESS trial also confirmed that various BP guided, fluid based protocols were ineffective in reducing mortality outcomes in septic shock from approximately 30 % [9.] So not only is BP the wrong measure for detecting fluid responsiveness, but it can only be applied in a small percentage of sick patients and has generally modest effectiveness in guiding the complex haemodynamics found in patients with sepsis.

Even if PPV had some applicability for identification of fluid responsiveness, the reliability and reproducibility of methods used for PPV measurements is poor. Pinsky’s own work comparing three different arterial pulse pressure methods (PiCCO, LiDCO, FloTrac) concluded that although they all generally generated approximately the same mean values, they often trended in different directions and could not be used interchangeably [10]. de Wilde was even more damning and after comparison of five arterial pulse pressure contour technologies (LiDCO, PiCCO, BMeye, Hemac) concluded that “none of the five pulse contour methods can replace thermodilution with four equally spread measures over the ventilator cycle, even after calibration by a precise thermodilution technique” [11]. These findings are further proof of the veracity of Guyton’s observations.

Given the crucial physiologic, technical and clinical limitations of PPV, why not really open our eyes, as Pinsky exhorts, and observe fluid responsiveness by directly measuring SV, and not the modulated surrogate BP? Well validated Doppler technologies accurately measure both SV and SVV, and are readily available to detect haemodynamic abnormalities in sepsis and guide fluid, inotropes and vasoactive therapies, and are noninvasive.

Doppler has an in vitro accuracy of 97 % measured against ultrasonic transit time flow probes [12, 13]. A comparison of transcutaneous Doppler (USCOM) with surgically implanted transit time flow probes in dogs, demonstrated a mean bias of 3 % and a precision of 13 % [14]. While a similar comparison of flow probes with USCOM in sheep over a sixfold CO range associated with inotrope and vasoactive therapy demonstrated sensitivity to 5 % changes in SV [15], while in humans this sensitivity is 7.5–10 %. This high sensitivity to SV change provides guidance of when to start fluid infusion, low SV and high SVV, and also when to stop, at the point of optimisation where additional fluid is no longer associated with an increased SV.

Transoesophageal Doppler monitoring has the best outcomes evidence of any technology for the management of perioperative fluid guidance, with a recent meta-analysis by the UK NHS Technical Adoption Centre reporting results from 10 RCTs and 2 nonrandomised outcomes trials that demonstrated improved mortality, morbidity and reduced costs using transoesophageal Doppler (CardioQ) for intra-operative fluid optimisation [16].

Combining accurate SV measures, in place of PPV, with the physiologic challenges recommended by Pinsky, results in a potent solution to the identification of cardiovascular reserve and fluid responsiveness, even in patients in whom PPV is otherwise excluded (AF, sepsis, on vasoactive therapies, free breathing etc.). Thiel et al. demonstrated a positive predictive value of 91 % for detection of fluid responsiveness in unselected ICU patients using USCOM measured SV changes associated with passive leg raising. Of the 102 patients studied, 61 % were diagnosed with sepsis, where both the SV and SVR can range from high, normal to low, 18 % had cardiac arrhythmias, 56 % were on vasopressors, 34 % were spontaneously breathing (66 % ventilated) [4]. In children Deep et al. [17] have identified novel haemodynamic features of sepsis and septic shock and modified fluid, inotrope and vasoactive therapy using USCOM flow based parameters to improve outcomes.

While we totally agree with Dr Pinsky, and Yogi Berra, that physiologic challenges are important tests to identify cardiovascular reserve, only when combined with an accurate measure of SV can we truly “see” the circulatory changes they induce. Non-invasive and accurate monitoring of SV at baseline and after intervention is the gold standard for effective and personalised haemodynamic monitoring. This physiologically rational approach is not just effective for the identification of fluid responsiveness, but also for detection of circulatory disease and guidance of inotropes and vasoactive therapies, and can be applied in adults and children across a range of diseases including hypertension, heart failure and sepsis.

With regard to the application of PPV for identification of fluid responsiveness, physiology and clinical evidence are in agreement with both recommending we look elsewhere, as Benes and Mahjoub concluded. While we agree with Yogi Berra, it was Sherlock Holmes who gave us the vital next clue for improving fluid management; “Watson, You see, but you do not observe. The distinction is clear” [18]. And it is elementary—direct SV monitoring is critical for improved management of fluid responsiveness.
